# Reproducible Ultrahigh SERS Enhancement in Single Deterministic Hotspots Using Nanosphere-Plane Antennas Under Radially Polarized Excitation

**DOI:** 10.1038/srep33218

**Published:** 2016-09-13

**Authors:** Jing Long, Hui Yi, Hongquan Li, Zeyu Lei, Tian Yang

**Affiliations:** 1 State Key Laboratory of Advanced Optical Communication Systems and Networks, Key Laboratory for Thin Film and Microfabrication of the Ministry of Education, UM - SJTU Joint Institute, Shanghai Jiao Tong University, Shanghai 200240, China

## Abstract

Surface enhanced Raman scattering (SERS) in a nanometer size hotspot has empowered the investigation of chemical structures and dynamic behaviors of one and a few molecules. However, further advancement is hindered by lack of large enough yet reproducible enhancement in single deterministic hotspots. To resolve this problem, here we introduce a nanosphere-plane antenna under radially polarized laser excitation experiment, which provides an electromagnetic enhancement of 10^**9~10**^ at the gap of each individual nanosphere-plane antenna and a root-mean-square error down to 10^**0.08**^ between them. The experiment also reveals a nonlinear SERS behavior with less than one plasmon, which is also observed within a single hotspot. The unprecedented simultaneous achievement of ultrahigh enhancement and reproducibility in deterministic individual hotspots is attributed to the combination of a well-controlled hotspot geometry, the efficient coupling between vertical antenna and laser which produces orders of magnitude higher enhancement than previous excitation methods, and low power operation which is critical for high reproducibility. Our method opens a path for systematic studies on single and few molecule SERS and their surface chemistry in an *in-situ* and well-controlled manner.

Surface enhanced Raman scattering (SERS) in nanoscale hotspots has been placed great hopes upon for identification of minimum chemical traces and *in-situ* investigation of single molecule structures and dynamics^1^,^2^,^3^,^4^,^5^,^6^,^7^. However, previous work consists of either high enhancement factors (EF) from random aggregates, or moderate EFs in deterministic devices, leaving observing the Raman spectra of single and few molecules in a reproducible manner a “hugely challenging task”^8^, as reviewed in the following. In the last decade, SERS detections of single small molecules in aggregates of metallic nanoparticles have been confirmed by the bi-analyte method, despite the extreme randomness of hotspot intensities and EFs^9^,^10^. It has been shown that the EFs vary from around 10^4^ to over 10^10^, with the 0.0003% most intense hotspots contributing 7% of the overall SERS signal^11^. It has also been pointed out that the most intense hotspots are required for detection of single small molecules with non-resonant Raman scattering cross sections as small as 10^−29^ to 10^−30^ cm^2^ sr^−1 ^12. To resolve the extreme randomness of EFs so as to achieve deterministic and systematic study of molecular dynamics, well-controlled fabrication of SERS substrates has been investigated extensively^8^,^13^,^14^,^15^,^16^,^17^. For example, electron beam writing of sub-5 nm gap optical antennas has been demonstrated by different groups recently, which nevertheless is no longer reproducible at such a small length scale^8^,^15^,^16^. An alternative approach that is directly related to our work in this paper is the nanoparticle-plane junction^2^,^5^,^18^,^19^,^20^,^21^,^22^,^23^,^24^, with reported experimental SERS EFs of an individual hotspot limited to about 10^5~8^, and with its reproducibility shown only after averaging tens or hundreds of hotspots. Meanwhile, there has been great progress in tip-enhanced Raman scattering (TERS) in recent years, with chemically mapping a single molecule and distinguishing adjacent molecules demonstrated at an unprecedented level^6^,^25^,^26^,^27^,^28^,^29^. However, TERS enhancement is mostly from the lightning-rod effect on its sharp metallic tip, while improving localized surface plasmon resonance (LSPR) contribution by modifying the TERS tip morphology could take a lot of efforts^6^, consequently TERS has been mostly used to detect single molecules that have large or resonant Raman scattering cross sections.

The lack of simultaneous ultrahigh enhancement and good reproducibility of SERS from single deterministic hotspots has severely limited systematic study of SERS at the single and few molecule level, and the promised applications of single molecule SERS, e.g. studies of *in-situ* molecular structures, single molecule dynamics, and chemical reactions at single molecule level, are far from being realized. To resolve this problem, here we present a method in which EFs as high as the most intense hotspots in previous work are achieved in a reproducible and well controlled manner, that is, electromagnetic EFs (EMEF) of 10^9~10^ with an root-mean-square (RMS) variation down to 10^0.08^. In this method, the vertically polarized LSPR of a single gold nanosphere on an atomically flat gold plane is excited by a radially polarized (RP) laser focal spot. A ~3 nm diameter hotspot is formed in the gap between the nanosphere and the plane with a monolayer of small molecules filling the gap, the Raman spectroscopy of which is detected. Our SERS EFs are several orders of magnitude higher than previous reports on nanoparticle-plane structures, since the LSPR excitation efficiency is greatly enhanced under the RP state of polarization by focusing the laser field to the vertical polarization which aligns with the orientation of the antenna. On the other hand, the LP state was commonly used in previous reports, in which the laser beam is either tilted, in a total-internal-reflection configuration, or scattered between the nanoparticles to produce a weaker vertically polarized field^2^,^5^,^18^,^19^,^20^,^21^,^22^,^23^,^24^. At the same time, the ultrahigh EFs allow all of our measurements to be best performed with laser powers lower than 1 μW for resonant Raman molecules, which is found to be important for obtaining stable SERS signals and high reproducibility. In addition, our experiment reveals that SERS EF has a nonlinear dependence on laser power with less than one plasmon, which can’t be explained by stimulated Raman scattering (SRS) as was previously hypothesized^6^,^29^, and which instead is an evidence of the recently proposed molecular cavity optomechanics model^30^,^31^.

## Results

**Figure 1:**
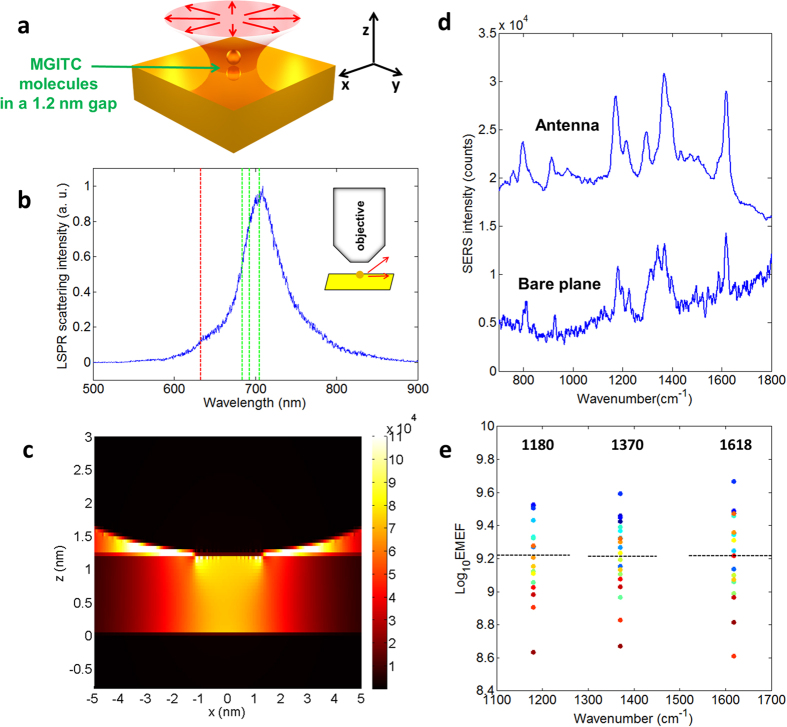
LSPR and SERS of antennas coated with a monolayer of MGITC. (**a**) Illustration of the gold nanosphere-plane antenna under RP excitation. The mirror image of the nanosphere is also plotted. (**b**) The LSPR spectrum of an antenna, by measuring its scattering outside of the NA of the illuminating objective. The nanosphere’s diameter is 60 nm. The laser wavelength and the three strongest Raman bands in the SERS experiment are labeled as red and green lines, respectively. (**c**) FDTD simulation of |*E*_*z*_^2^| in the antenna’s junction gap, normalized by the |*E*_*z*_^2^| of an incident p-wave at its resonance wavelength 691 nm. (**d**) The SERS spectrum of an antenna (lifted up by 10000 counts), and that of a monolayer of MGITC on a bare gold plane (background subtracted by 20000 counts). The laser power at sample is 300 nW for the antenna, and 1.5 mW for the bare plane. The integration time is 4 s for the antenna, and 10 s for the bare plane. (**e**) SERS EMEFs of twenty antennas for three Raman bands at 1180 cm^−1^, 1370 cm^−1^ and 1618 cm^−1^. Each three dots with the same color come from one same antenna. The dashed lines are the average EMEFs for each band.

A schematic illustration of our method is shown in Fig. 1a. A chemically synthesized 60 nm gold nanosphere is on top of a 200 nm thick atomically flat gold plane. The nanosphere pairs with its mirror image to form a vertically oriented and vertically polarized optical antenna. An RP He-Ne laser beam at 633 nm is focused by an objective with a numerical aperture (NA) of 0.9 to excite the nanosphere-plane antenna^32^. A monolayer of probe Raman molecules is coated on the surface of the nanosphere whose Raman scattering is collected by the same focusing objective.

The LSPR spectrum of one of the antennas is shown in Fig. 1b. The laser wavelength and three of the strongest Raman peaks of MGITC at 1180, 1370 and 1618 cm^−1^ are labeled to show that they all fall within the LSPR resonance. In Fig. 1c, FDTD simulation shows a 7 × 10^4^ fold increase of the vertical electric field intensity, |*E*_*z*_^2^|, in the junction gap hotspot on LSPR resonance, according to classical electromagnetics. A gap height of 1.2 nm and a nominal refractive index of 1.5 have been used in the simulation to represent a monolayer of MGITC on top of the gold plane^33^. The simulated hotspot intensity spectrum is close to the experimental scattering spectrum and shown in Supplementary Fig. S1. Since the size and wavelength of the illuminating focal spot are nearly an order of magnitude larger than the antenna, it is appropriate to consider the antenna as a point. Therefore the response of the antenna to external illumination, including the electrical field enhancement and the LSPR mode, is assumed not to change with the structure of the illumination focal spot to simplify our theoretical study. Note that, in the experiment a monolayer of molecules coated on the nanosphere is used instead of on top of the gold plane, but we simulate the latter due to the difficulty to finely construct a 1.2 nm thin spherical shell in a square simulation mesh with limited computer memory.

Figure 1d shows the SERS spectrum of an antenna under a laser power of 300 nW at sample and an integration time of 4 s. To calculate the EF, the Raman spectrum from a monolayer of MGITC coated on a bare atomically flat gold plane under the same RP laser focal spot is also shown. The EMEFs of twenty different antennas for three of the strongest Raman peaks are plotted in Fig. 1e. The EF is defined by comparing with an imaginary experiment in which the molecule is measured in air using a linearly polarized (LP) laser beam and the same focusing objective. Details of EF calculation are described in Supplementary Methods. In the calculation, the hotspot area, *A*_hotspot_, is taken to be 9.3 nm^2^ according to the FDTD result, which will be discussed later. We attribute the calculated EF to electromagnetic effects, since in both the antenna experiment and the bare gold plane experiment, the thiol group (-SH) of MGITC forms a covalent bond with the gold surface so that they are expected to have chemical EFs close to each other. That being said, it has been shown by Ikeda *et al.* that the chemical EF varies with the crystalline direction of the gold face to which the molecules are bound^20^, which may change our estimation of the EMEF value by up to a few times. According to Fig. 1e, the EMEFs of the three Raman bands at 1180, 1370 and 1618 cm^−1^ are 10^9.22±0.23^, 10^9.21±0.22^ and 10^9.22±0.24^, respectively. The first number on the exponent is the average value of Log_10_ EMEF, and the second number is the RMS error. These results show both ultrahigh enhancement and reasonable reproducibility. All of the twenty SERS spectra are shown in Supplementary Fig. S3.

**Figure 2:**
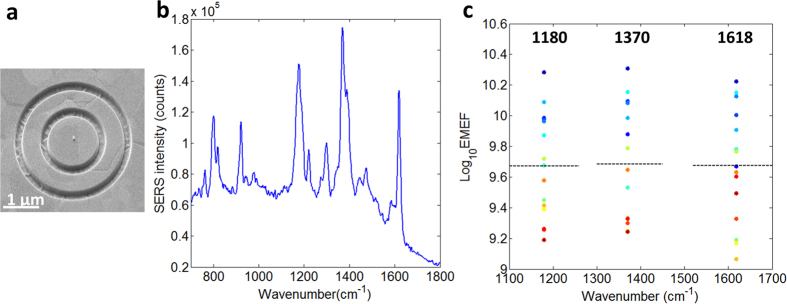
SERS of antennas coated with a monolayer of MGITC and enclosed by double rings. (**a**) A scanning electron micrograph of the device. (**b**) A typical SERS spectrum. The laser power at sample is 300 nW. The integration time is 4 s. (**c**) SERS EMEFs of fifteen antennas for three Raman bands at 1180 cm^−1^, 1370 cm^−1^ and 1618 cm^−1^. Each three dots with the same color come from one same antenna. The dashed lines are the average EMEFs for each band.

Further, to collect the part of Raman scattering that couples to propagating surface plasmon polaritons (SPP), another fifteen antennas each enclosed by 100 nm deep double rings were measured. The rings were fabricated around the nanospheres by dual-beam focused ion beam (FIB) milling. A scanning electron micrograph (SEM) of the device, a typical SERS spectrum, and the EMEF distribution are shown in Fig. 2. The rings are intentionally made wide enough so as not to be covered by the laser focal spot, so that the nanospheres are excited by the laser in the same way as the ones without rings. By FDTD simulation, for a vertical point dipole at 692.9 nm, that is, the 1370 cm^−1^ Raman band, which is 31 nm above a gold plane, 62.9% of its output power is carried by SPPs, 28.1% by far-field radiation, and the rest 9% by lossy surface waves. Adding the double rings scatters 69.8% power of SPPs to far-field radiation. However, in our device, the double ring structure has a Purcell effect that reduces the dipole’s coupling to SPPs to 55.1% of that without rings. The combination of all these effects, together with the interference between the dipole far-field radiation and the double-ring far-field scattering of SPPs, improves the collection efficiency of dipole output power by a NA = 0.9 objective from 21.4% to 66.2%, and improves the collected power with the same dipole moment by a factor of 2.22. Experimentally, as shown in Fig. 2c, higher EMEFs of the same three Raman bands have been obtained, which are 10^9.67±0.33^, 10^9.68±0.36^ and 10^9.67±0.36^. The average EMEF values can be further improved by adjusting the distance between the nanosphere and the rings to make use of the Purcell effect to generate more SPPs. The alignment error between the antennas and the double rings contribute to increase of the EMEF errors. All of the fifteen SERS spectra are also shown in Supplementary Fig. S3.

Excluding chemical enhancement, the SERS EFs in our experiments are comparable to the highest in previous reports on random aggregates^3^,^7^,^11^,^12^. In addition, thanks to the low laser power, the SERS signals were stable for more than five minutes without any obvious evidence of molecule degradation.

**Figure 3:**
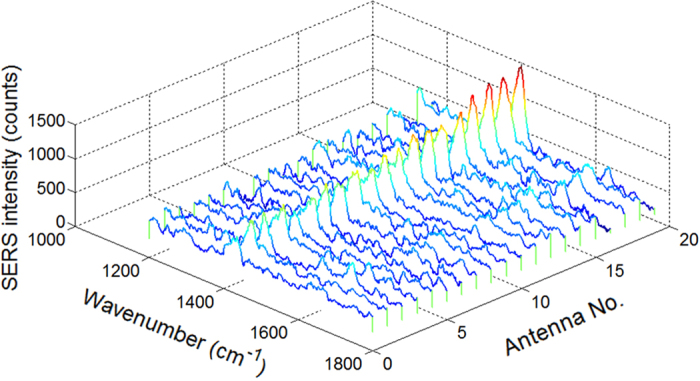
SERS of twenty antennas coated with a monolayer of 4NBT. The laser power at sample is 300 nW. The integration time is 4 s. The background fluorescence from impurities has been subtracted for three of the antennas.

In the above, we have used MGITC as the probe molecule due to its large resonant Raman scattering cross section at the He-Ne laser wavelength, so that the Raman signals from the bare gold plane can be measured to calculate EMEF. In order to confirm the capability of our method to detect non-resonant Raman scattering from small molecules, we repeated the measurement for twenty antennas coated with a monolayer of 4NBT molecules, with the results shown in Fig. 3. Not only can we observe clear SERS signals from the –NO_2_ stretching mode at 1336 cm^−1^ under 300 nW laser power and 4 s integration time, but a considerably better reproducibility than that of MGITC which is 10^±0.08^. We suppose the higher reproducibility to be the consequence of the molecules’ better chemical stability when they are non-resonant with the laser^34^, less chemical EF dependence on the gold crystal face and having a higher density of smaller molecules in each hotspot.

**Figure 4:**
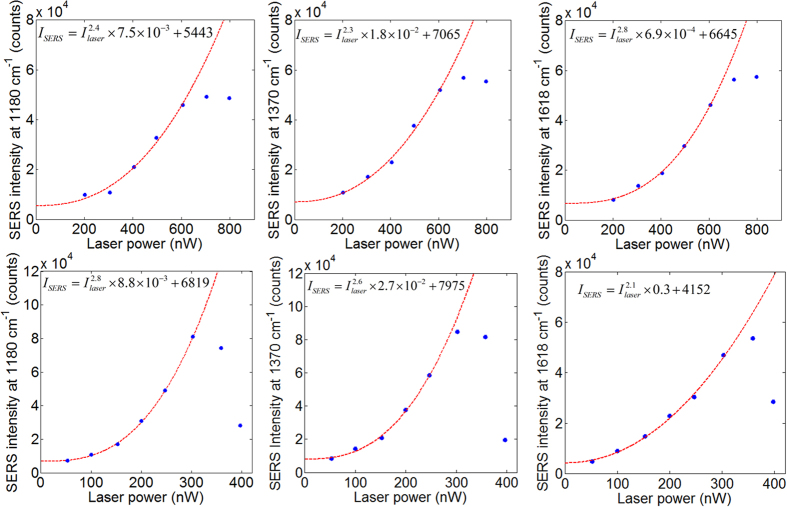
SERS intensity versus laser power. First row: The SERS intensities of three Raman bands of MGITC versus laser power at sample, for an antenna coated with a monolayer of MGITC. Second row: The same as the first row, but for an antenna enclosed by double rings. The blue dots are measurement results. The red dashed curves and the equations in the figure are exponential fitting results. The integration time is 4 s. The SERS intensity measurement has an average RMS error around 6–7% before rolling over.

The value of *A*_hotspot_ needs further discussion. Values from less than 1 nm^2^ to several tens of nm^2^ have been used in the literature, while each MGITC molecule occupies an area of 0.6 × 1 nm^2 ^33. Ultra-small hotspots seem to be evidenced by ultrahigh resolution TERS mapping experiments, both under ultrahigh vacuum and low temperature and in ambient conditions^6^,^28^,^29^. The sub-nm TERS hotspots were related to the discovery of nonlinear dependence of TERS intensity on laser power, which was suggested to result from SRS^6^,^29^. In our experiment, a strong nonlinear dependence of the SERS intensity on the laser power has also been observed, as shown in Fig. 4. Here we can exclude the possibility of SRS effect since there is much less than one plasmon on average in the antenna, as explained in the following. 300 nW at 633 nm corresponds to 9.5 × 10^11^ photons per second, and the plasmon life time in the antenna is 5.3 fs according to the LSPR bandwidth in Fig. 1b, so that no more than an average of 5.0 × 10^−3^ plasmons are confined in the antenna under 300 nW laser power. Therefore SRS by the plasmons is much weaker than the spontaneous Raman scattering^35^. More recently, a molecular cavity optomechanics model was proposed to explain both the nonlinearity and ultrahigh resolution^30^. Compared with the planar antenna example calculated in that model, in our experiment, the tighter hotspot confinement and larger Raman activity of resonant Raman molecules shall allow us to observe dynamical backaction amplification of molecular vibrations with much less plasmons in the antenna. Further, the exponential fittings of the nonlinear SERS behavior in Fig. 4 don’t start from the origin, which is a sign of more complicated mechanism due to quantization of molecular vibration^31^.

## Discussion

Compared with previous work on nanoparticle-plane junction SERS, the enormously improved EFs and reproducibility in our experiment come from three factors. First, the RP laser beam is critical for obtaining maximum |*E*_*z*_| in the laser focal spot and consequently efficient excitation of the vertical antennas and ultrahigh EFs. In general, the laser beam and the focusing element should have the same central symmetry in order to couple to vertical LSPR. However, previous methods used an anti-central-symmetric LP laser beam and a central-symmetric microscope objective, and the mismatch of symmetry was compensated by inclining the illumination which was proved inefficient in ref. 36. The theoretical and experimental vectorial profiles of LP and RP focal spots are compared in Supplementary Fig. S4 to illustrate the vast differences between |*E*_*z*_| in the focal spots with different states of polarization. Theoretically, in an ideal RP focal spot with an NA of 0.9, 50% of the total beam power is contained in the vertically polarized field^37^. The |*E*_*z*_^2^| is further improved by the mirror reflection by a factor of around 4, so that the extinction cross section of |*E*_*z*_^2^| by the nanoparticle-plane antenna can be around four times as much as that of the s-polarized illumination by a nanoparticle dimer. Consequently, an around 50% extinction efficiency of the RP focal spot on LSPR resonance is estimated in our experiments, i.e. nearly all of the |*E*_*z*_^2^| is scattered or absorbed, which is reduced as the laser wavelength is blue tuned off the LSPR resonance.

Second, the atomically flat gold plane is critical for reproducibility, while most previous work used evaporated or sputtered metal films which had nanometer scale surface roughness. The variation of EF in our experiment is largely because the nanospheres are actually polyhedrons with crystal plane facets, so that different nanospheres can have different interfaces with the hotspots by contacting the plane with a facet, an edge or an apex^38^,^39^,^40^. Supplementary Fig. S5 shows a transmission electron micrograph (TEM) of the polyhedron, and the LSPR spectra of twenty antennas in which variation caused by interface coupling effects is obvious. In addition, variations in the diameter (±8% according to BBI Solutions) and geometry of the nanospheres can also contribute to the variation of LSPR spectra.

**Figure 5:**
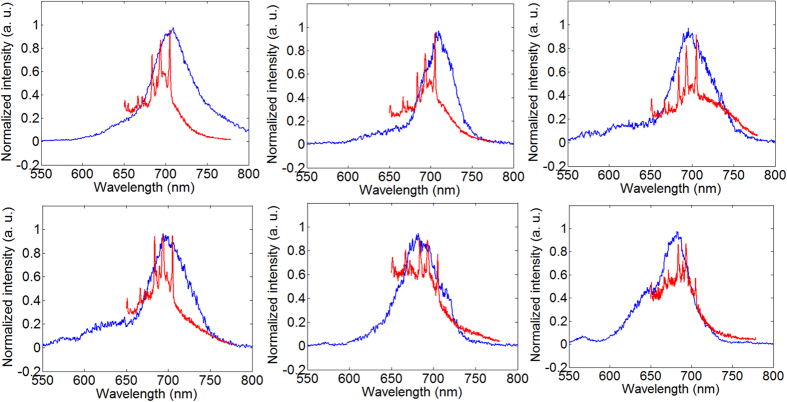
LSPR and SERS spectra of six antennas coated with a monolayer of MGITC. Blue: LSPR. Red: SERS.

It is interesting to observe that, in our experiment, there is a good correlation between the LSPR spectra and the intensity ratios between the three major SERS bands. In Fig. 5, each sub-figure contains the LSPR spectrum and SERS spectrum of the same antenna, with only those antennas with a close-to-Lorentzian LSPR spectral profile being plotted. According to the figure, it is obvious that as the LSPR shifts from shorter to longer wavelength, the three major SERS bands are enhanced in a corresponding order of shorter to longer wavelength. This explains that, in both Figs 1e and 2c, the EMEFs of the three Raman bands are quite close to each other after averaging an ensemble of antennas, while they can be much more different for a single antenna. The correlation between LSPR and SERS is in contradiction to a general observation for noble metal nanoparticle aggregate samples, in the latter of which single hotspot SERS enhancement is broadband and unrelated to LSPR^41^.

In addition, it has been shown by Ikeda *et al.* that the chemical EF is dependent on the crystalline direction of gold, and they have achieved reproducible SERS by averaging hundreds of nanoparticle-plane junctions on the same gold crystal face^20^. At last, since we are only concerned with the enhancement of electric field along the vertical polarization in this work, there is no molecular orientation dependence of the EF we have calculated, but rotation and diffusion of molecules at the interface can also contribute to EF variation.

Third, the EFs roll over irreversibly at sub-μW laser powers, and the reproducibility significantly worsens in and near the roll-over regime as shown in Fig. 4, due to molecule bleaching, drift, rotation and desorption. Therefore low laser power operation is critical. The EFs of our experiment are just high enough for us to operate with low enough power so as not to degrade the MGITC SERS intensities from any hotspots, and at the same time to obtain an enough signal-to-noise ratio for single hotspot observation. From this perspective, to achieve reproducible SERS detection in nanoparticle-plane junctions with previous excitation methods is also much more difficult due to their limited EFs. Further study on single hotspots’ roll-over behavior is a meaningful subject. In fact, it is widely accepted that photobleaching in SERS is a poorly understood phenomenon, while it significantly affects understanding and application of SERS^42^,^43^. It is more appropriate to analyze the roll-over behavior on a single hotspot basis, since each hotspot rolls over at a different threshold laser power with a different speed even in our system with high EF reproducibility.

In conclusion, with a new excitation scheme of focusing a sub-μW RP laser beam onto a gold nanosphere - atomically flat gold plane antenna sandwiching a monolayer of molecules, we have obtained ultrahigh SERS EFs that are quite uniform between different hotspots. This method opens a path for deterministic and systematic studies of nanoscale molecular behaviors by Raman spectroscopy^44^,^45^. An example of using this method for monitoring single molecule level chemical events at an unprecedented level are reported by refs 46 and 47. In the future, we will place the nanosphere at the tip of an atomic force microscope to make a precisely controlled junction gap to accommodate single molecules. In addition, we expect this method to provide a sensitive and reproducible tool to explore the physics of nanoscale hotspots. For example, it allows studying SERS nonlinearity^6^,^29^,^32^ with less than one plasmon, and at the same avoiding laser heating and bleaching effects to obtain less complicated experimental results at room temperature. Recently SERS probe has been used to measure nonlocality and quantum tunneling effects, for which our method could be useful as well^16^,^48^,^49^.

## Materials and Methods

### Sample preparation

#### (1) Antennas coated with a monolayer of malachite green isothiocyanate (MGITC) molecules

First, 13.5 μL of 45 μM MGITC (Invitrogen M689) ethanol solution and 1 mL of 5.2 × 10^9^/mL gold nanosphere ultra-purified water solution (BBI Solutions, 60 nm mean diameter, ±8% variation) were incubated together for 2 hours at room temperature^18^. Then the functionalized gold nanosphere solution was 1:1 diluted with ultra-purified water, and drop-casted onto the gold planes. The gold planes are 200 nm thick Au (111) films on mica substrates (PHASIS), which have been deposited by magnetron sputtering and then hydrogen flame annealed to obtain atomically flat surfaces. Next the samples were rinsed with ultra-purified water and dried under a stream of nitrogen.

#### (2) Bare gold plane coated with a monolayer of MGITC molecules

To compare with the antennas, bare gold plane samples were first immersed in 1 μM MGITC ethanol solutions for 10 minutes, then rinsed with ethanol and dried under a stream of nitrogen^33^.

#### (3) Antennas coated with a monolayer of 4-nitrobenzenethiol (4NBT) molecules

First, 0.5 mL of 6.5 × 10^9^/mL gold nanosphere ultra-purifed water solution was added to 0.5 mL of 4 μM 4NBT (Sigma-Aldrich) ultra-purifed water solution and mixed for 2 hours at room temperature. Then the functionalized gold nanosphere solution was drop-casted onto the gold planes. Next the samples were dried under a stream of nitrogen.

### Optical setup and spectroscopy measurement

#### (1) Raman scattering measurement

A He-Ne laser working at 632.8 nm and TEM_00_ mode was used to excite the molecules. The laser beam passed through a liquid crystal polarization converter (ARCoptix) and was converted to an RP state of polarization. The RP laser beam was focused on to each antenna on the sample through a long working distance 100× Plan Apo objective, whose NA is 0.9. The laser power at sample was measured right after the focusing objective with a silicon photodiode. Reflection from the sample, including Raman scattering, was collected by the same objective, passed through a long-pass filter, and detected by a monochromator installed with an electron multiplying CCD (EMCCD) detector.

#### (2) LSPR spectra measurement

A super continuum source was focused onto each antenna through the same 100× objective. The scattered light was collected outside the NA of the objective with a lens whose NA is 0.15, as shown in the inset of Fig. 1b. The collecting lens focused the scattered light into a fiber-bundle directed to the monochromator and EMCCD detector. The power of the super continuum source was carefully decreased by neutral density filters in order not to damage the samples.

#### (3) Identification of nanospheres

The small scattering cross section of the antennas and the large reflection off the gold plane render it extremely difficult to find the nanospheres under optical microscopes without special methods. The same 100× objective was used as part of a home-built microscope to observe the nanospheres. A spatial filter blocked the central part of the objective’s entrance pupil so that the nanospheres were illuminated at an inclined angle. The nanospheres appear as dark spots on a bright background, due to the antennas’ absorption and scattering of the inclined illumination. In addition, position markers were made by FIB milling on the gold planes prior to nanosphere distributions, and SEM images were taken before the SERS experiment to compare with the optical microscopy images, so that the nanospheres can be identified repeatedly. It is important to limit the SEM imaging dose upon the antennas so as not to damage them and to mitigate carbon deposition.

### Reproducibility characterization

We have selected those gold nanoparticles with spherical shapes under SEM for optical experiments. Around 10% of the gold nanoparticles have irregular non-spherical shapes. Otherwise, we have not intentionally excluded any nanospheres for SERS EF reproducibility characterization.

### Finite difference time domain (FDTD) simulation

FDTD simulations were carried out to calculate the LSPR spectrum and the hotspot intensity profile of the antenna, using Lumerical FDTD Solutions. The nanosphere-plane antenna is excited by a broadband total-field scattered-field source, which is a p-polarized planewave at 30°-to-normal incidence. The boundary conditions are perfectly matched layers except for one mirror symmetry plane across the center of sphere. The finest grid size of the mesh is 0.05 nm in and near the junction gap, and increases to 4 nm at away from the junction gap.

## Additional Information

**How to cite this article**: Long, J. *et al.* Reproducible Ultrahigh SERS Enhancement in Single Deterministic Hotspots Using Nanosphere-Plane Antennas Under Radially Polarized Excitation. *Sci. Rep.* **6**, 33218; doi: 10.1038/srep33218 (2016).

## Supplementary Material

Supplementary Information
