# Supplementary material for: Reproducible Ultrahigh SERS Enhancement in Single Deterministic Hotspots Using Nanosphere-Plane Antennas Under Radially Polarized Excitation
Source: Sci Rep. 2016 Sep 13;6:33218. doi: 10.1038/srep33218 (PMC5020428; doi:10.1038/srep33218)
Supplement: Supplementary Information [file srep33218-s1.doc]

**Reproducible Ultrahigh SERS Enhancement in Single Deterministic Hotspots Using Nanosphere-Plane Antennas Under Radially Polarized Excitation**

Jing Long, Hui Yi, Hongquan Li, Zeyu Lei and Tian Yang

1. **Hotspot intensity spectrum by FDTD simulation**


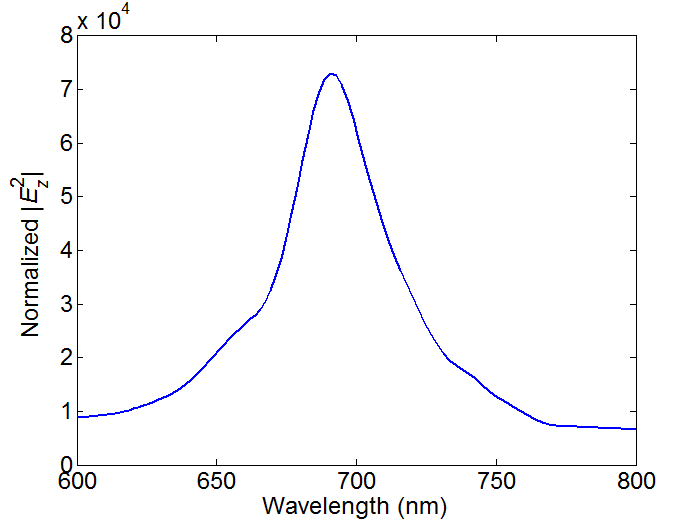


**Figure S1 FDTD simulation of |*E****_z_***^2^| at the center of the antenna’s junction gap.** The antenna in Fig. 1. is used. The illumination is an around 30°-to-normal *p*-polarized broadband planewave. The intensity is normalized by the |*E_z_*^2^| of illumination.

1. **Supplementary Methods: EMEF calculation**

The EMEFs of our experiments are calculated by equation (1).

$$\begin{matrix} EMEF=\frac{I_{\text{SM-hotspot}}}{I_{\text{SM-air}}} \\ \begin{matrix} \end{matrix}\begin{matrix} \begin{matrix} \end{matrix} \end{matrix}\begin{matrix} \end{matrix}\begin{matrix} \end{matrix}\begin{matrix} \end{matrix}=\frac{P_{\text{laser2}}}{P_{\text{laser1}}}\cdot\frac{I_{\text{hotspot}}/\mathrm{A}_{\text{hotspot}}}{I_{\text{plane}}/\mathrm{A}_{\text{RP}}}\cdot2^{4}\cdot\frac{\left|E_{\text{RP}}^{2}\right|}{\left|E_{\text{LP}}^{2}\right|}\begin{matrix} \begin{matrix} \begin{matrix} & \begin{matrix} \begin{matrix} & \end{matrix} & \end{matrix} & \end{matrix} & (1) \end{matrix} & \end{matrix} \end{matrix}$$

The first line of equation (1) is the definition of SERS EF, or the definition of EMEF when the chemical contribution to EF is excluded as in this paper. *I*_SM-hotspot_ is the collected Raman scattering from a single molecule in the junction gap hotspot. *I*_SM-air_ is the collected Raman scattering in an imaginary experiment where an LP laser beam with the same power is focused onto a single molecule in air, using the same focusing and collecting objective as in our experiment. The second line is how we calculate the EMEF. *P*_laser1_ is the laser power for exciting the molecules in the hotspot, which is 300 nW. *P*_laser2_ is the laser power for exciting the molecules on the bare gold plane, which is 1.5 mW. *I*_hotspot_ and I_plane_ are the collected Raman scattering from a monolayer of molecules in the hotspot and on the bare gold plane divided by their respective integration time. *A*_hotspot_ and *A*_RP_ are the full-width-half-maximum (FWHM) area of the hotspot and the laser focal spot, respectively, so that *I*/*A* is proportional to Raman scattering power per molecule. Note that *A*_hotspot_ is the area of |*E*^4^| since SERS EMEF in an LSPR hotspot is proportional to |*E*^4^|, while *A*_RP_ = 0.08 μm^2^ is the area of |*E*^2^| since Raman is a linear process by itself [1,2]. Due to the mirror effect of the gold plane, the transverse *E* is weak and only *E_z_* is considered in the estimation of *A*’s. The 2^4^ factor counts for the EM enhancement contributed by the bare gold plane compared to the imaginary in-air experiment, which includes the mirror effect which increases the excitation |*E_z_*^2^| by a factor of around 2^2^, and the Purcell effect which increases the Raman emission rate by another factor of around 2^2^. |*E*_RP_^2^|/|*E*_LP_^2^|equals the ratio between the |*E*^2^| values at the center points of the RP and LP focal spots, which is theoretically calculated to be 0.89. A line-scanning of the FDTD simulation result of the hotspot (Fig. 1c) is shown in Fig. S2, which gives a FWHM hotspot diameter of 3.45 nm and *A*_hotspot_ of 9.3 nm^2^.


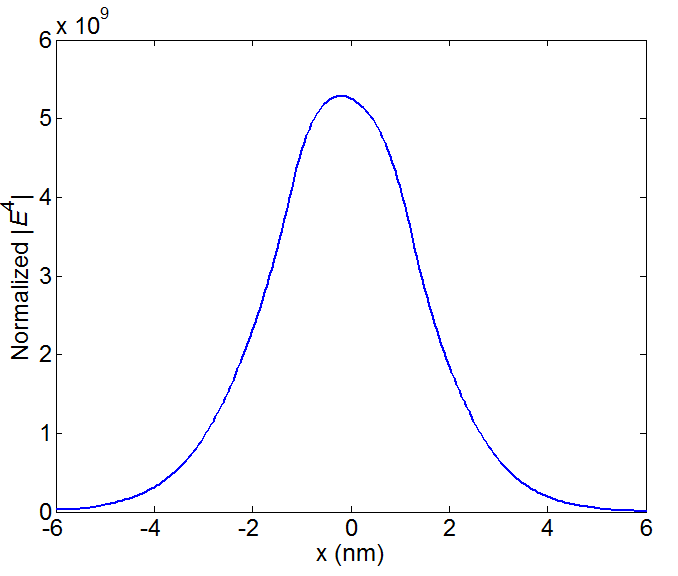


**Figure S2 Line scanning of |*E*^4^| across the center of the hotspot.** FDTD simulation result is plotted. The **|***E*^4^**|** value has been normalized to the incident field.

1. **SERS of multiple antennas coated with monolayer MGITC**


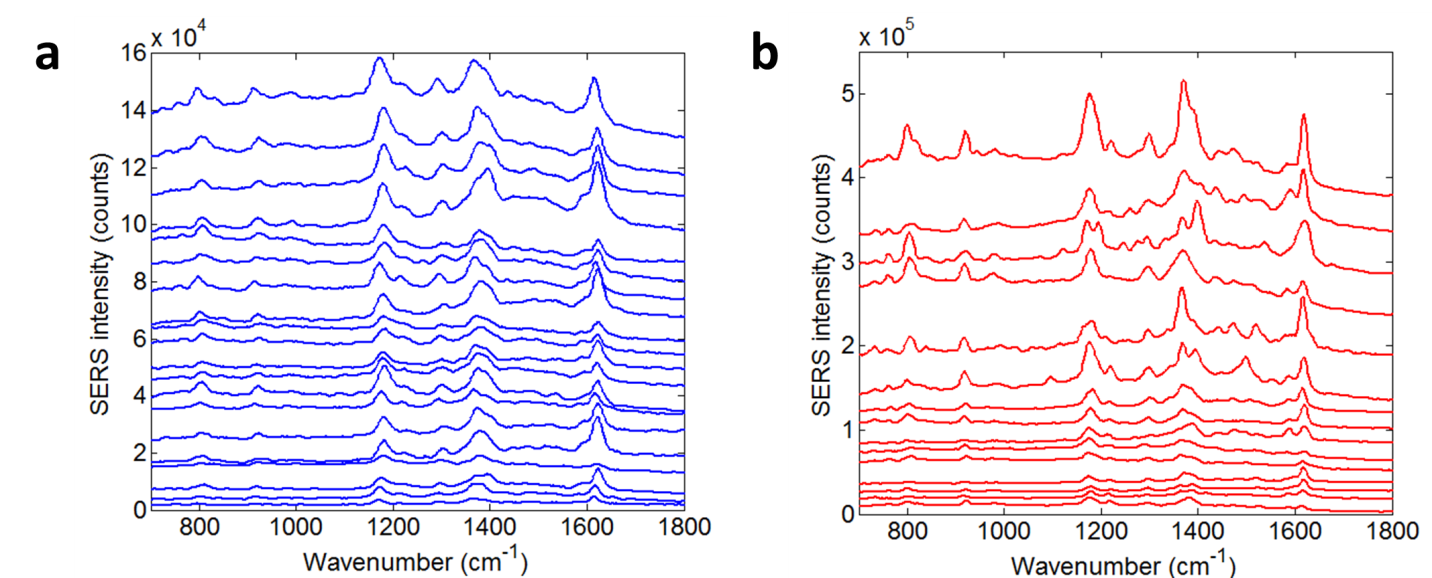


**Figure S3**. **SERS spectra of multiple antennas coated with a monolayer of MGITC**. The laser power at sample is 300 nW. The integration time is 4 s. Each spectrum has been lifted to a different height in the figures. **a**, Twenty antennas. **b,** Fifteen antennas with double rings

1. **Comparison between RP and LP focal spots**

RP and LP laser beams pointing in the *z* direction are focused through an objective with NA=0.9. The vectorial profiles of the focal spots are experimentally characterized by raster scanning a gold nanosphere on a silica aerogel substrate and measuring the scattered far field, following Ref [3]. The theoretical calculation results are also presented, following Ref [4]. The vectorial profiles shown in Fig. S4 indicate much larger |*E_z_*^2^| in the RP focal spot than in the LP focal spot.


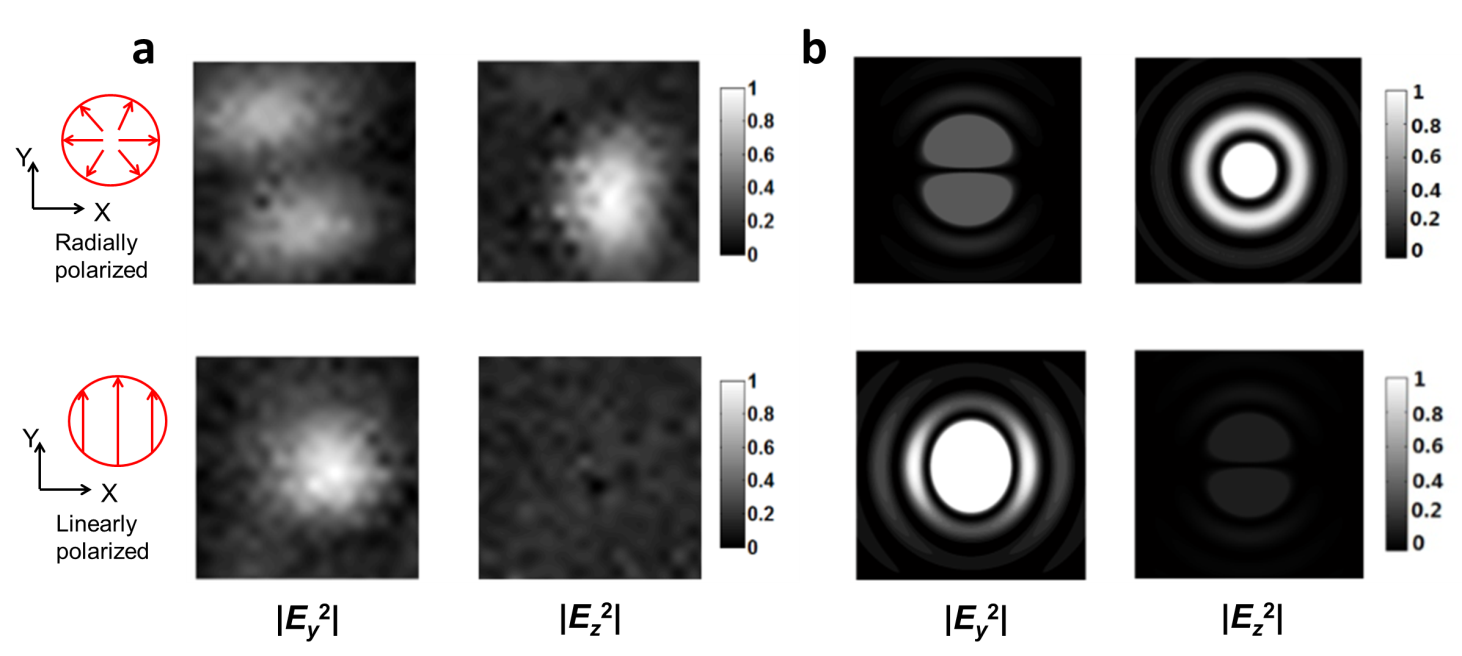


**Figure S4 Vectorial profiles of RP and LP focal spots.** The first row is RP focal spot profiles. The second row is LP focal spot profiles. Each column is the profile of either *|E_y_*^2^| or *|E_z_*^2^|, as labeled. Laser wavelength is 632.8 nm. Focusing objective NA=0.9. All images are 1.2×1.2 μm^2^. **a**, Experimental results. **b**, Theoretical results. The intensities are normalized to the maximum *|E*^2^| of the respective focal spots.

1. **Effects of nanosphere-plane interfaces**

The TEM image of a 60 nm gold nanosphere coated with a monolayer of MGITC is shown in Fig. S5a. It is a polyhedron. The contact between the polyhedron and the plane may be in the form of facet, edge or apex. We have observed significant deviation between the LSPR spectra of different antennas, as shown in Fig. S5b and c. Three out of twenty antennas show double peaks in their LSPR spectra, as the orange curve in Fig. S5c. This is reported to result from strong charge concentration and plasmon coupling at the junction, which is sensitive to the morphology of the interface [5,6]. On the other hand, the high reproducibility of SERS EFs implicates that the values of *A*_hotspot_ may not vary a lot between different antennas.


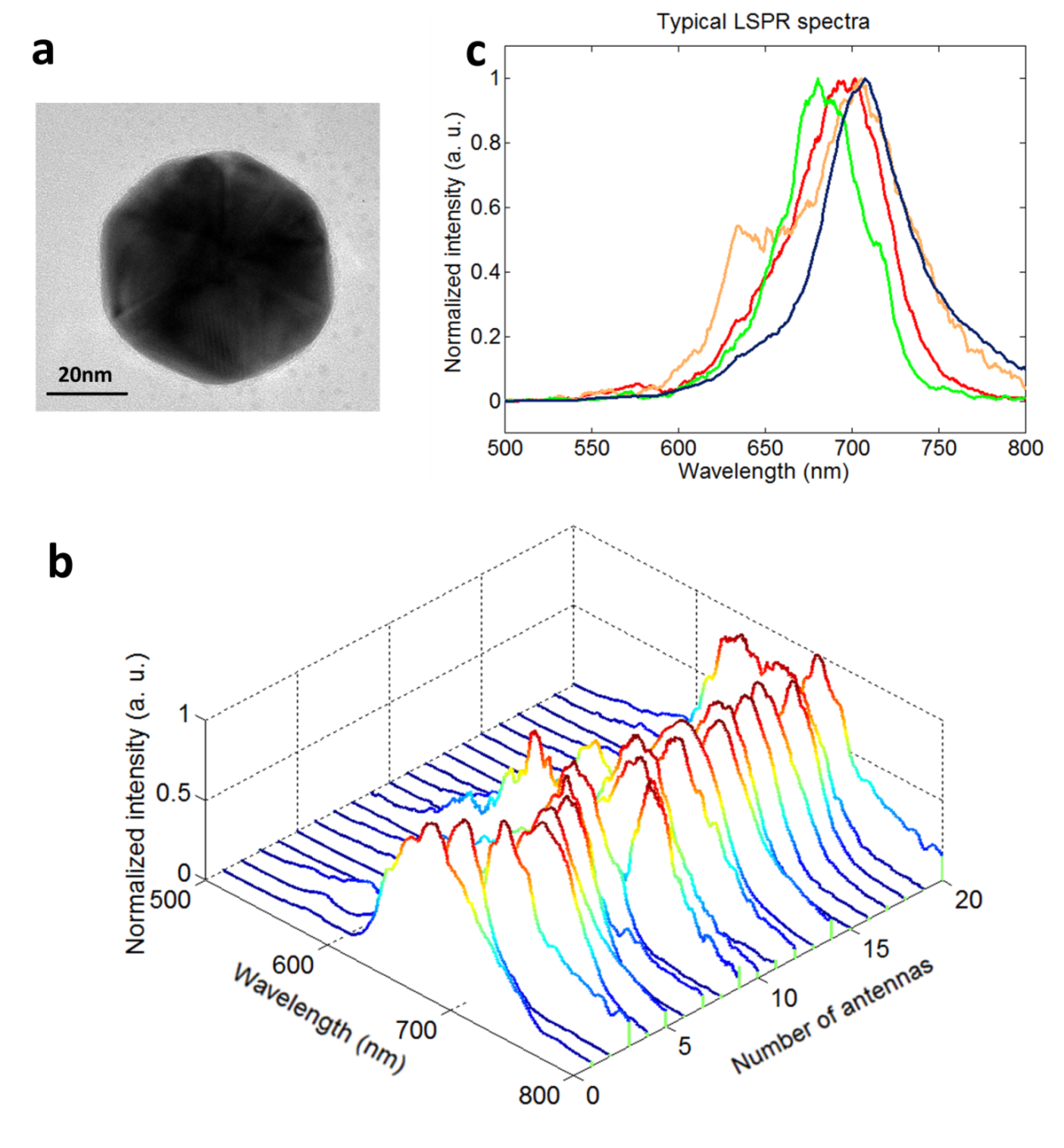


**Figure S5** **a**, TEM image of a 60 nm gold nanosphere coated with a monolayer of MGITC. **b**, LSPR spectra of twenty antennas, normalized to the same height. These antennas are the same as in Fig. S3a. **c**, Some representative LSPR spectra from **b**.

**References**

1. Wang, D., Yang, T. & Crozier, K. B. Optical antennas integrated with concentric ring gratings: electric field enhancement and directional radiation. *Opt. Express* **19,** 2148-2157 (2011).
2. Liu, B., Wang, D., Shi, C., Crozier, K. B. & Yang, T. Vertical optical antennas integrated with spiral ring gratings for large local electric field enhancement and directional radiation. *Opt. Express* **19,** 10049-10056 (2011).
3. Yi, H. , Long, J., Li, H., He, X. & Yang, T., Scanning metallic nanosphere microscopy for vectorial profiling of optical focal spots. *Opt. Express* **23,** 8338-8347 (2015).
4. Novotny, L. & Hecht, B. L. Propagation and focusing of optical fields. *Principles of Nano-optics* Ch.3 (Cambridge Univ. Press, Cambridge, 2006).
5. Mertens, J. et al. Controlling subnanometer gaps in plasmonic dimers using graphene. *Nano Lett*. **13,** 5033-5038 (2013).
6. Romero, I., Aizpurua, J., Bryant, G. W. & García De Abajo, F. J. Plasmons in nearly touching metallic nanoparticles: singular response in the limit of touching dimers. *Opt. Express* **14,** 9988-9999 (2006).
